# Prediction of COVID-19 Severity Using Chest Computed Tomography and Laboratory Measurements: Evaluation Using a Machine Learning Approach

**DOI:** 10.2196/21604

**Published:** 2020-11-17

**Authors:** Daowei Li, Qiang Zhang, Yue Tan, Xinghuo Feng, Yuanyi Yue, Yuhan Bai, Jimeng Li, Jiahang Li, Youjun Xu, Shiyu Chen, Si-Yu Xiao, Muyan Sun, Xiaona Li, Fang Zhu

**Affiliations:** 1 Department of Radiology The People's Hospital of China Medical University & The People's Hospital of Liaoning Province Shenyang China; 2 Department of Pulmonary and Critical Care Medicine Shengjing Hospital of China Medical University Shenyang China; 3 Department of Gastroenterology Shengjing Hospital of China Medical University Shenyang China; 4 Department of Intensive Care Unit The People's Hospital of Yicheng City Yicheng China; 5 The First Clinical Department China Medical University Shenyang China; 6 The Second Clinical Department China Medical University Shenyang China; 7 Department of Radiology The People's Hospital of Yicheng City Yicheng China; 8 Department of Laboratory Medicine The People's Hospital of Yicheng City Yicheng China; 9 Intanx Life (Shanghai) Co, Ltd Shanghai China; 10 School of Fundamental Sciences China Medical University Shenyang China; 11 Department of Cardiovascular Ultrasound The People's Hospital of China Medical University & The People's Hospital of Liaoning Province Shenyang China

**Keywords:** COVID-19, severe case prediction, computerized tomography, machine learning, CT, scan, detection, prediction, model

## Abstract

**Background:**

Most of the mortality resulting from COVID-19 has been associated with severe disease. Effective treatment of severe cases remains a challenge due to the lack of early detection of the infection.

**Objective:**

This study aimed to develop an effective prediction model for COVID-19 severity by combining radiological outcome with clinical biochemical indexes.

**Methods:**

A total of 46 patients with COVID-19 (10 severe, 36 nonsevere) were examined. To build the prediction model, a set of 27 severe and 151 nonsevere clinical laboratory records and computerized tomography (CT) records were collected from these patients. We managed to extract specific features from the patients’ CT images by using a recently published convolutional neural network. We also trained a machine learning model combining these features with clinical laboratory results.

**Results:**

We present a prediction model combining patients’ radiological outcomes with their clinical biochemical indexes to identify severe COVID-19 cases. The prediction model yielded a cross-validated area under the receiver operating characteristic (AUROC) score of 0.93 and an F_1_ score of 0.89, which showed a 6% and 15% improvement, respectively, compared to the models based on laboratory test features only. In addition, we developed a statistical model for forecasting COVID-19 severity based on the results of patients’ laboratory tests performed before they were classified as severe cases; this model yielded an AUROC score of 0.81.

**Conclusions:**

To our knowledge, this is the first report predicting the clinical progression of COVID-19, as well as forecasting severity, based on a combined analysis using laboratory tests and CT images.

## Introduction

In December 2019, an epidemic of pneumonia caused by a newly identified coronavirus (SARS-CoV-2) emerged in China and has been spreading worldwide ever since [1]. According to the World Health Organization, to date, the COVID-19 pandemic has affected more than 200 countries worldwide, causing global panic and contributing to fears of market recession and mass unemployment. The novel virus causing COVID-19 was identified to have originated from the Orthocoronavirinae subfamily, the same subfamily as SARS-CoV and MERS-CoV [2], and it was thus officially named SARS-CoV-2. This virus might invade the human airway epithelial cells by binding to the angiotensin-converting enzyme 2 receptor (ACE2), in a mechanism similar to that of SARS-CoV [3,4].

The clinical features of COVID-19 are atypical, ranging from mild systematic symptoms, including intermittent fever (83%) and lower respiratory tract reactions such as cough (61%), to less common ones such as shortness of breath (14.5%), muscle ache (18.6%), headache (11.8%), and diarrhea (6.1%) [1,5]. Some patients with COVID-19 might develop severe complications such as acute renal failure (2.1%), acute respiratory distress syndrome (ARDS, 8.9%), or shock (2.2%), and some might even die (3.7%) [1,6]. The clinical and epidemiological spectrum of COVID-19 is quite diverse and is still not fully understood. Previous reports have suggested that the whole world’s population is generally prone to COVID-19 [7]. Nevertheless, older patients who have underlying diseases such as cerebral infarction, chronic obstructive pulmonary disease, bronchiectasis, or diabetes are more prone to severe pneumonia, respiratory failure, septic shock, or even death caused by multiple organ failure [6].

SARS-CoV-2 is highly infectious and can be primarily transmitted through direct or indirect contact, droplets, and aerosol. Diagnosis of COVID-19 usually involves a combination of the patient’s travel history, clinical symptoms, and radiological and biochemical findings. Patchy ipsilateral pulmonary consolidations are visible on a computerized tomography (CT) scan initially, during the early course of COVID-19. As the infection progresses, the consolidations are reduced and appear as bilateral ground-glass opacities, marking the prominent radiological features of COVID-19 [8]. The “white lung” radiograph, a characteristic finding suggesting that the patient urgently requires oxygen inhalation, has only been observed in a few critical patients with ARDS [9-11]. Other biochemical index changes associated with a COVID-19 diagnosis include lymphopenia, increased C-reactive protein and lactate dehydrogenase (LDH) levels, and thrombocytopenia [5].

Antiviral medication and glucocorticoids are most commonly used for the clinical treatment of COVID-19, with antibacterial medication sought when bacterial co-infection is detected [12]. Given the insufficient clinical trial data for the safety and efficacy of remdesivir and chloroquine, there is still no persuasive evidence for effective medicine for the treatment of COVID-19 [13]. It is noteworthy that approximately 11.5% of all reported patients with COVID-19 developed severe illness characterized as ARDS. These patients were transferred to an intensive care unit, as they required mechanical ventilation and even extracorporeal membrane pulmonary oxygenation (ECMO), the efficacy of which is very limited according to a retrospective study, wherein 5 of 6 patients receiving ECMO eventually died [14,15]. In fact, the mortality rate of severely ill patients with a confirmed diagnosis of COVID-19 is 60%, indicating the importance of early detection and prediction of COVID-19 severity [14,15]. However, at present, it is a critical challenge to identify a patient with COVID-19 who might require intensive care before certain clinical symptoms are observed. Therefore, there is an urgent need to develop an effective prediction or forecasting model for patients with COVID-19.

Our study aimed to address this challenge: we developed a prediction model for COVID-19 clinical progression, by combining radiological outcome based on CT scans with biochemical indexes. To extract essential features from CT scans, we segmented the lungs from the CT volumetric images by using a deep convolutional neural network (CNN). Finally, we also developed a model to forecast COVID-19 severity based on the results of the patients’ laboratory tests before the patients were classified as severe cases. To our knowledge, this is the first study to report a prediction model for assessing COVID-19 severity by combining radiological outcomes with clinical biochemical indexes. We believe that our prediction model will shed light on predicting disease severity for all patients with COVID-19.

## Methods

### Patient Information

We collected samples from 46 patients who visited People’s Hospital of Yicheng City between January 16, 2020, and March 4, 2020, and were diagnosed with COVID-19 according to the Chinese Government Diagnosis and Treatment Guideline (Trial 5th version; Medicine, 2020). For a confirmed diagnosis of COVID-19, nucleic acid was extracted from sputum or throat swab samples using a nucleic acid extractor (EX3600, Shanghai Zhijiang Biotechnology Co.) and a nucleic acid extraction reagent (No. P20200201, Shanghai Zhijiang Biotechnology Co.).

Fluorescence-based quantitative polymerase chain reaction (PCR; ABI7500) and SARS-CoV-2 nucleic acid detection kit (triple fluorescence PCR, No. P20200203, Shanghai Zhijiang Biotechnology Co.) were used for nucleic acid detection. This kit uses a one-step reverse transcription–PCR combined with Taqman technology to detect RNA-dependent RNA polymerase (*RdRp*), envelope (E), and nucleocapsid (N) genes. PCR results were concluded to be positive if (1) *RdRp* gene was positive (cycle threshold [Ct]<43) and either E or N gene was positive (Ct<43), or (2) if two sequential tests for *RdRp* were positive and those for E and N genes were negative. The 46 study patients with COVID-19 were classified into 2 types: (1) nonsevere, comprising patients showing mild symptoms without radiological manifestations of pneumonia, fever, or respiratory tract symptoms with radiological manifestations of pneumonia, and (2) severe, comprising patients meeting any of the following criteria—respiratory rate ≥30 breaths/min, pulse oxygen saturation ≤93% in resting state, partial pressure of arterial oxygen ≤300 mm Hg (1 mm Hg=0.133 kPa), respiratory failure requiring mechanical ventilation, shock incidence, and admission to intensive care unit with other organ failure. In total, 10 patients were categorized as severe cases and 36, as nonsevere cases. The last follow-up of these patients was on March 10, 2020.

### Ethics Approval

Approval for studies on CT screening and clinical test results was obtained from the Medical Ethics Committee of The People’s Hospital of Yicheng City, China (2020Yc002)

### Data Collection

We collected and reviewed clinical information of 46 patients with COVID-19 after admission, including clinical signs and symptoms, comorbidities, travel history, laboratory tests, and CT scans. To consolidate all patients’ records into a single table, missing records for a given day were noted as “NA” (not available). In all, we obtained 178 records (27 severe and 151 nonsevere cases) from 105 different laboratory tests and chest CT images. Note that throughout the clinical course, each patient had more than one record variably classified as severe or nonsevere. Patients with at least one severe record were classified as severe cases.

### Data Processing and Statistical Analysis

We identified 44 laboratory tests that had more than 50% missing values (NA), and we then imputed the NAs with the mean values. Related laboratory tests were identified based on the criterion that the *P* value (Mann-Whitney U test) between the severe and nonsevere groups is smaller than .05. In all, we found that 36 laboratory tests were related to the detection of COVID-19 severity. The patients’ CT images were processed using a pretrained CNN with a U-Net structure [16] to segment the lung lobes from the background. The intensities were then normalized to grayscale for all patients before further analysis. We then analyzed the intensities of the 3D CT volumes within lung masks to obtain CT features for each record.

### Severity Prediction Models

Prediction models were developed to predict patient severity based on laboratory and CT signatures collected at corresponding dates. Each patient record was considered a sample for a model; as a result, 178 samples were evaluated using those models. Before using model prediction, we used random forest importance score, mutual information, and fold change as possible approaches to select important model features while avoiding potential overfitting. We found mutual information to be the most robust approach. We considered different candidate machine learning models, including random forest classifier, gradient boost classifier, XGB classifier, logistic classifier, and supported vector machine. Random forest was found to be the best classifier, and model parameters were optimized using a genetic algorithm (Tree-Based Pipeline Optimization Tool). The area under the curve of the receiver operating characteristic (AUROC) and F_1_ scores were used to evaluate model accuracy considering the dataset imbalance. All models were trained with 5-fold cross-validation with stratified train-test splits that preserve the percentage of samples in severe and nonsevere groups. All cross-validated results were averaged over 20 runs.

### Severity Forecasting Models

Forecasting models were built to forecast patient severity based on laboratory and CT signatures collected from nonsevere cases at admission. In these models, instead of the patients’ records, the patients themselves were considered as samples to build forecasting relationships. CT records were not collected as frequently as laboratory tests were performed, and initial, nonsevere CT records were not available for 3 severe cases. Therefore, we built two separate random forest models based on CT features and laboratory tests with 7 and 10 severe cases, respectively. Other model details were identical to those of the severity prediction models.

## Results

### Overview of Study Patients

We collected clinical data of 46 patients with COVID-19 who were admitted at the People’s Hospital of Yicheng City, between mid-January and early-March 2020. We recorded 305 biochemical test results from 105 different tests, based on the clinical reports of all 46 study patients (Multimedia Appendix 1). General patient information is shown in Table 1. The general trend that older patients with COVID-19 tend to develop more systemic symptoms was not observed in our study [17]. However, patients with comorbidities, especially diabetes and hypertension, tended to develop more severe symptoms than others. Moreover, patients with severe COVID-19 typically experienced fatigue, anorexia, malaise, chest congestion, and shortness of breath.

**Table 1:** Characteristics and symptoms of study patients.

Characteristic			Values		
			All cases (N=46)	Severe cases (n=10)	Nonsevere cases (n=36)
Age in years, mean (range)			48.8 (24-71)	56.8 (33-71)	46.5 (24-71)
**Sex, n (%)**					
	Male		25 (54)	6 (60)	19 (53)
	Female		21 (46)	4 (40)	17 (47)
**Exposure, n (%)**					
	Wuhan		24 (52)	5 (50)	19 (53)
	Family		4 (9)	2 (20)	2 (6)
	Community		5 (11)	0 (0)	5 (14)
	None		13 (28)	3 (30)	10 (28)
**Comorbidity, n (%)**					
	Hypertension		11 (24)	5 (50)	6 (17)
	Cardiovascular disease		6 (13)	2 (20)	4 (11)
	Chronic liver disease		3 (7)	2 (20)	1 (3)
	Diabetes		5 (11)	3 (30)	2 (6)
	Leukoderma		1 (2)	0 (0)	1 (3)
	Chronic kidney disease		1 (2)	0 (0)	1 (3)
	Hyperuricemia		1 (2)	0 (0)	1 (3)
	Chronic lung disease		2 (4)	0 (0)	2 (6)
**Symptoms** **, n (%)**					
	Dry Cough		28 (61)	6 (60)	22 (61)
	Cough with phlegm		9 (20)	2 (20)	7 (19)
	**Fever**				
		High	8 (17)	3 (30)	5 (14)
		Mid	20 (43)	4 (40)	10 (28)
		Mild	14 (30)	3 (30)	17 (47)
	Fatigue		25 (54)	9 (90)	16 (44)
	Anorexia		33 (72)	9 (90)	24 (67)
	Malaise		34 (74)	10 (100)	24 (67)
	Headache		7 (15)	3 (30)	4 (11)
	Nausea		1 (2)	0 (0)	1 (3)
	Diarrhea		5 (11)	2 (20)	3 (8)
	Dyspnea		1 (2)	1 (10)	0 (0)
	Chest congestion		16 (35)	5 (50)	11 (31)
	Shortness of breath after activity		19 (41)	6 (60)	13 (36)

In all, 52% (24/46) patients had a travel history to or from Wuhan within the past 1 month, and 20% (9/46) patients had clear exposure history in the local city (Table 1). According to the patients’ medical records, 80% of the severe cases had one or more comorbidities, whereas only 34% of the nonsevere cases had comorbidities. This finding is consistent with that of previous studies [18]. Moreover, 81% (37/46) patients had cough and only 20% (9/46) patients reported sputum production. Fever was the most common symptom; however, severe cases (7/10, 70%) had a higher proportion of mid- to high-grade fever (ie, >38.9°C) than the nonsevere cases (15/36, 42%). More than half of the patients (25/46, 54%) experienced fatigue, and approximately three-quarters of them had anorexia (33/46, 72%) or malaise (34/46, 74%); these symptoms were observed in almost all severe cases (fatigue, 9/10, 90%; anorexia, 9/10, 90%; and malaise, 10/10, 100%). Headache, nausea, diarrhea, and dyspnea were rarely observed in both severe and nonsevere cases. Moreover, less than half of all patients reported chest congestion (16/46, 35%) or shortness of breath after activity (19/46, 41%), and these symptoms were approximately 20% more common in severe cases than in nonsevere cases.

### Prediction Based on Laboratory Tests

**Figure 1:**
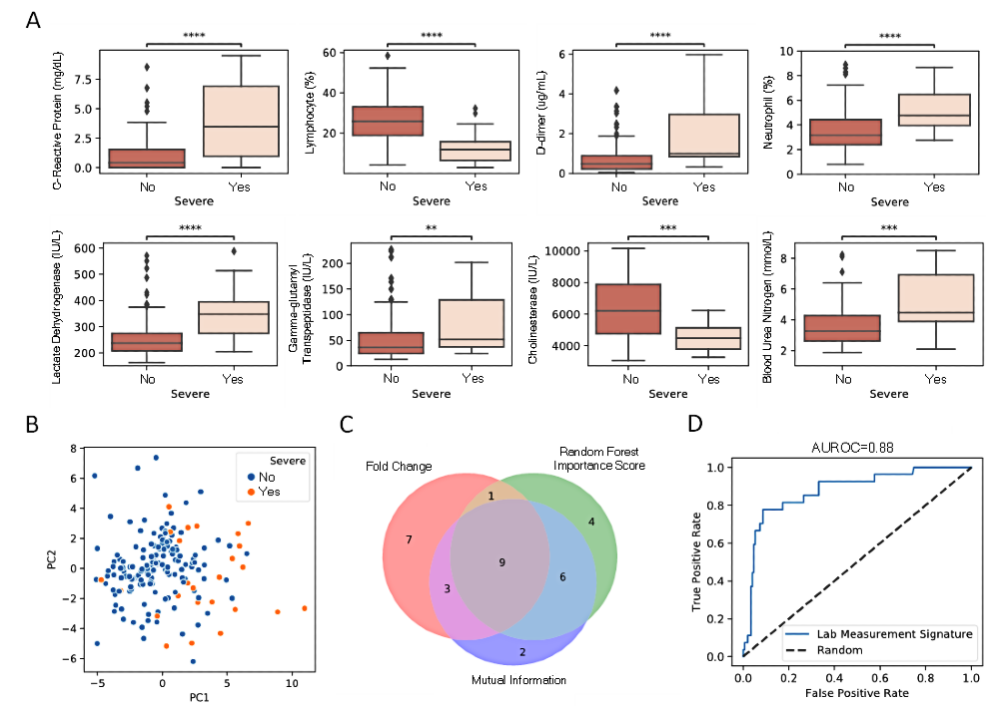
Correlation of laboratory tests with COVID-19 severity. (A) Top-8 laboratory tests ranked by fold change. (B) Principal component (PC) analysis of all laboratory tests. (C) Venn diagram of the top features selected by 3 different approaches: random forest importance score, mutual information, and fold change. (D) Area under receiver operating characteristic of classification using a signature of 12 laboratory tests. The asterisk annotations denote the following: * 1.00e-02<P≤5.00e-02, ** 1.00e-03<P≤1.00e-02, *** 1.00e-04<P≤1.00e-03, **** for P≤1.00e-04.

Data processing yielded 61 laboratory tests results, 36 of which were significantly related to severity. Eight related laboratory tests that showed the largest fold change are illustrated in Figure 1A. Among these tests, D-dimer, LDH, and lymphocytes were found to be associated with mortality risk [17]. Principal component analysis results clearly showed separation between the severe and nonsevere groups, indicating that the COVID-19–related laboratory tests can be used to identify disease severity (Figure 1B). To build a statistical model to predict severity, we first selected the most important laboratory tests to avoid overfitting. Three different approaches—fold change, random forest importance score, and mutual information—were considered the top-ranking laboratory features. In fact, the three approaches led to very similar ranking, and the top features obtained from mutual information resulted in the largest intersection with those obtained from the other two approaches (Figure 1C). This finding suggests that mutual information is the most robust feature selector among the three abovementioned approaches; therefore, we used mutual information to select laboratory features to be used in the model. We used a random forest model with hyperparameters optimized by a genetic algorithm (see Methods) to predict severity based on laboratory features. Our results suggested that the prediction accuracy does not further increase with an increase in the number of laboratory features beyond 12. As a result, a signature of the top 12 laboratory features was considered, and the corresponding model yielded a cross-validated AUROC score of 0.88 and an F_1_ score of 0.69 (Figure 1D).

### Extraction of CT Features

**Figure 2:**
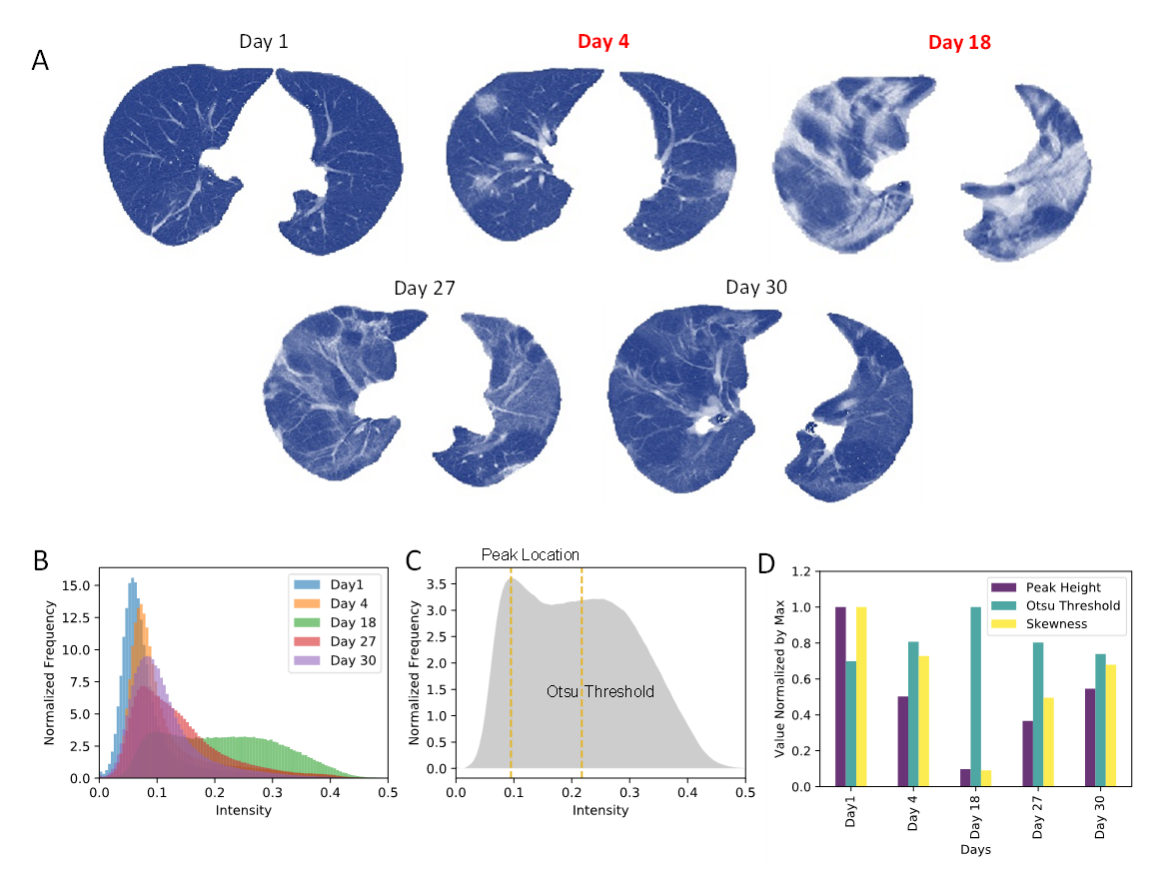
Computed tomography (CT) feature extraction. (A) Segmented lung images from the middle CT slice for a patient with a full course of COVID-19 from nonsevere to severe and then from severe to nonsevere. The patient’s severe records are presented in red color. (B) Intensity histograms of the volume CT within segmented lung masks for five consecutive records of the patient. (C) Peak location and Otsu threshold features from the intensity histogram on Day 18. (D) Variation of 3 different CT features along the course of the disease.

**Figure 3:**
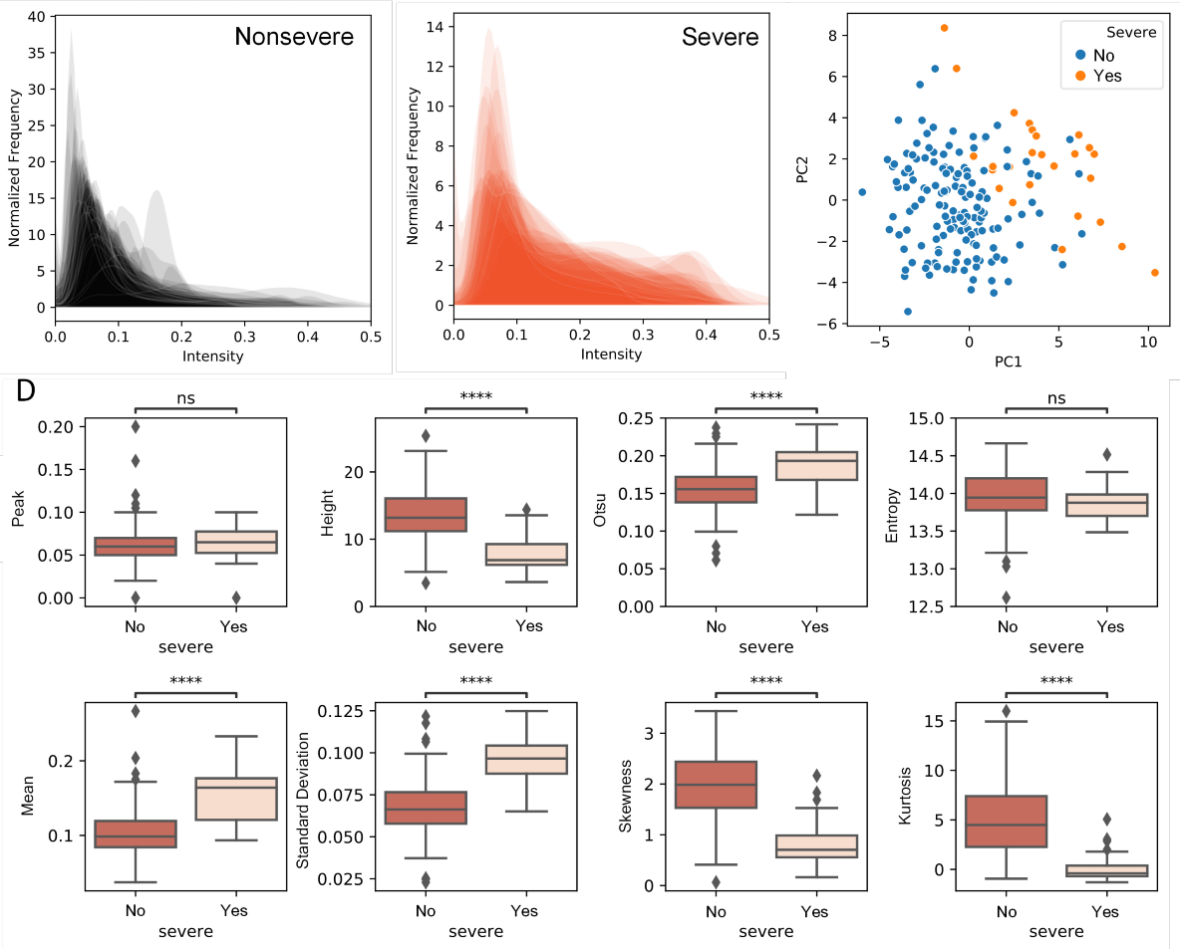
Computed tomography (CT) intensity distribution and extracted features of patients with COVID-19. (A) Intensity distribution of CT volumes from nonsevere cases. (B) Intensity distribution of CT volumes from severe cases. (C) Principal component analysis of all CT features. (D) All CT features between severe and nonsevere groups. “Peak” stands for peak location, and “height” stands for peak height. The asterisk annotations denote the following: * 1.00e-02<P≤5.00e-02, ** 1.00e-03<P≤1.00e-02, *** 1.00e-04<P≤1.00e-03, **** P≤1.00e-04.

To extract CT features, we first segmented the lungs from the CT volumetric images using a deep CNN, U-Net. Because the CNN was pretrained with several annotated datasets, including a COVID-19 dataset from MedSeg [16], we directly transferred the trained CNN to segment CT images of the study patients. The CT slices acquired across a clinical course of a patient are shown in Figure 2A. Right after onset, the patient was diagnosed with nonsevere disease, with no apparent opacity visible in lung CT. The patient was classified as severe on Day 4, and this continued for 2 weeks thereafter, with increasing amounts of ground-glass opacity and patchy consolidation. The ground-glass opacity and consolidation started to fade away from Day 27, and on Day 30, the patient was confirmed to be asymptomatic. As seen in Figure 2A, the opacity of the segmented lung lobes is associated with disease severity. The opacity can be represented by the intensity distribution within the segmented lung volumes (Figure 2B). Note that only the slices in the middle are shown in Figure 2A for illustration purposes; all slices were considered, however, to determine intensity distribution. As the symptoms became severe, the background became increasingly opaque, as indicated by the peak locations and peak heights of the intensity distribution. The distribution also changed from unimodal to bimodal. Therefore, we considered peak location and height as well as the first four moments of the intensity distribution (ie, mean, standard deviation, skewness, and kurtosis) as CT features. Since the intensity distribution can become bimodal, we also added the Otsu threshold to reflect the bimodality and entropy to supplement standard deviation. Three exemplary CT features observed along the clinical course of the patient are shown in Figure 2D. Thus, Otsu threshold is an excellent predictor for severity based on visualization. We then analyzed all 178 CT records and determined the corresponding intensity distributions (Figure 3A and 3B). We found that distributions of severe and nonsevere cases were in direct contrast in terms of peak height and skewness. Principal component analysis also showed improved separation between the 2 groups (Figure 3C). Among the 8 CT features examined, peak location and entropy were not significantly related to severity, whereas all the other 6 CT features showed a statistically significant relation (Figure 3C). Mean and standard deviation, as well as skewness and kurtosis, were highly correlated; therefore, standard deviation and kurtosis were not considered as CT features.

### Prediction Based on CT and Laboratory Features

**Figure 4:**
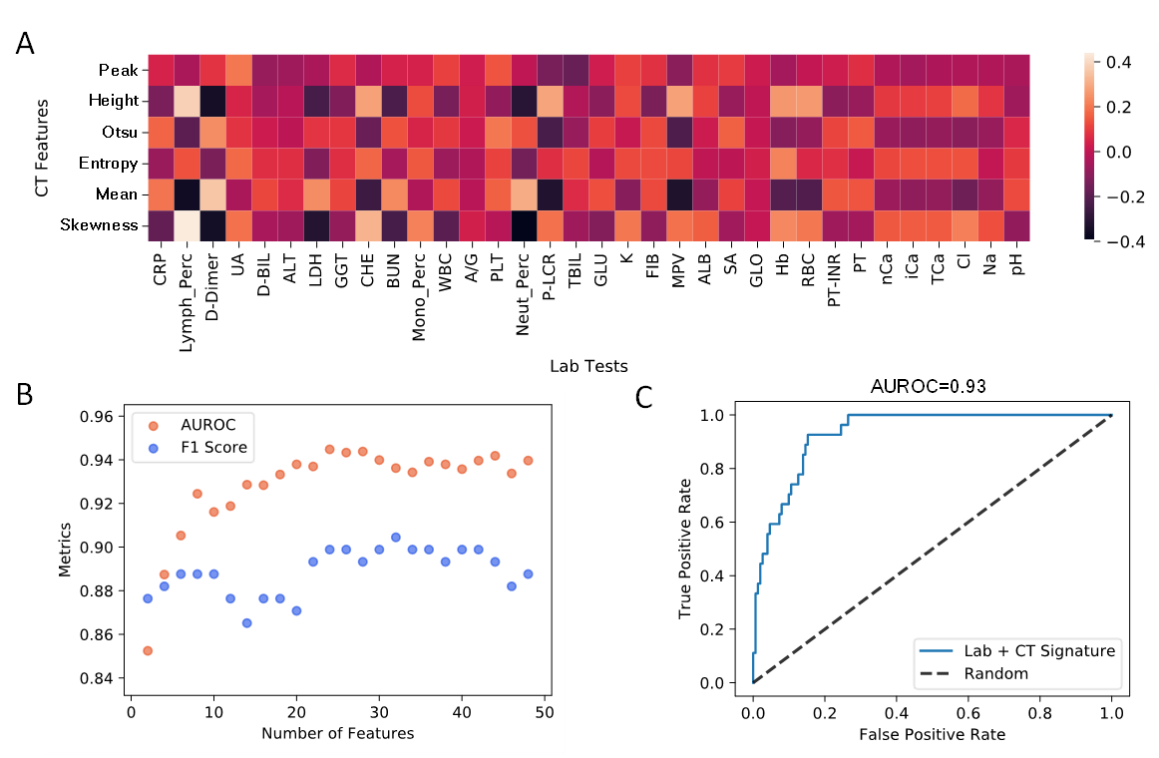
Prediction based on computed tomography (CT) and laboratory features. (A) Spearman correlation heatmap between CT and laboratory features. “Peak” stands for peak location, and “height” stands for peak height. A summary table describing all CT and laboratory features and their abbreviations is provided in Multimedia Appendix 2. (B) Model accuracy metrics with an increased number of features. (C) Area under receiver operating characteristic of classification using a signature of 15 CT and laboratory features.

The CT feature extraction enables quantitative prediction with signatures of both CT and laboratory features. We first analyzed the Spearman correlation between the CT and laboratory features (Figure 4A). Most features were not significantly correlated; however, lymphocyte, neutrophil, D-dimer, and platelet–large cell ratio showed good correlation with several CT features. Similarly, we used mutual information to select features to be used in the model. We used a random forest model with optimized hyperparameters to predict severity from CT and laboratory features. We selected a signature of 16 features from the feature number analysis (Figure 4B). The corresponding prediction model yielded a cross-validated AUROC score of 0.93 and an F_1_ score of 0.81 (Figure 4C), which are considerably improved from the corresponding scores of the model with laboratory tests only (Figure 1D). The signature includes CT peak height, CT intensity mean, CT intensity skewness, CT Otsu threshold, lymphocyte percentage, gamma-glutamyl transpeptidase, LDH, C-reactive protein, white blood cell, D-dimer, cholinesterase, neutrophil percentage, hemoglobin, tricyclic antidepressant, albumin, and chloride.

### Forecasting Disease Severity

**Figure 5:**
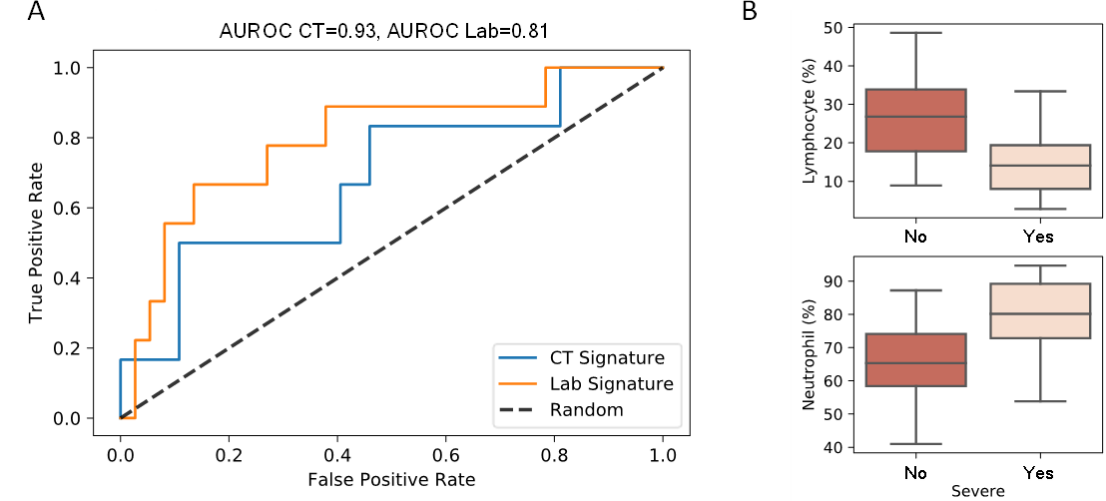
COVID-19 severity forecasted using the prediction model. (A) Forecasting severity using patient’s nonsevere records noted upon admission. (B) Laboratory tests showing a significant relation to the severity forecast.

Forecasting disease severity has significant clinical importance, as it allows clinicians to better prepare for treatment course. In addition to predicting severity based on CT and laboratory signatures, we also developed a statistical model to forecast severity from patient records upon admission when they were considered nonsevere. Although CT features are excellent predictors of severity, they are not as good for forecasting, yielding an AUROC of 0.68. In contrast, the random forest model based on laboratory tests yielded an AUROC of 0.81, indicating excellent forecasting predictability (Figure 5A). Other metrics considered for forecasting are presented in Table 2. This statistical model comprised 8 laboratory tests, among which lymphocyte and neutrophil counts (percentage) showed the highest fold change. In addition to comorbidity, we identified 8 laboratory tests that could be used for severity forecasting: individual counts of lymphocyte, neutrophil, monocyte, and eosinophil; red blood cell distribution width; hemoglobin; procalcitonin; and platelet–large cell ratio.

**Table 2:** Metrics of prediction and forecasting models. Mean and standard deviation values across 5 cross-validation splits are shown. AUROC: area under the receiver operating characteristics.

Features	Prediction model		Forecasting model	
	Laboratory only, mean (SD)	Laboratory and CT^a^, mean (SD)	CT only, mean (SD)	Laboratory only, mean (SD)
Precision	0.75 (0.2)	0.82 (0.05)	0.55 (0.22)	0.61 (0.23)
Recall	0.7 (0.15)	0.79 (0.1)	0.56 (0.23)	0.61 (0.11)
AUROC^b^ Score	0.86 (0.1)	0.93 (0.03)	0.68 (0.22)	0.81 (0.14)
F_1_ Score	0.69 (0.17)	0.81 (0.05)	0.56 (0.22)	0.60 (0.16)
Accuracy	0.87 (0.04)	0.88 (0.03)	0.78 (0.12)	0.83 (0.06)

^a^CT: computed tomography.

^b^AUROC: area under the receiver operating characteristic.

## Discussion

In this study, we collected clinical records from 46 patients with COVID-19 (27 severe and 151 nonsevere records) and developed a prediction model using a combination of radiological outcomes and clinical biochemical indexes, to identify disease severity. Using the model thus developed, we successfully achieved an AUROC score of 0.93 to identify the patient’s severity status. Furthermore, we established a model for forecasting disease severity based on the combined features recorded before the patients were classified as severe cases, resulting in an AUROC score of 0.81.

In the history of confrontation between human beings and pathogens, humans have always been prone to losing the battle when the development of effective medicine or vaccine is extremely difficult owing to the high variability of the pathogenic genome, such as in the case of influenza virus, HIV, or SARS. Even though the reported mortality rate of COVID-19 (1.4% [5]) is not as high as that of SARS (10% [19]), individuals with underlying health conditions such as hypertension, cardiovascular disease, chronic kidney disease, and diabetes (2.89-, 3.84-, 2.22-, and 2.65-fold higher risk, respectively [20]) are much more vulnerable to COVID-19. Approximately half of the patients with COVID-19 are above 50 years of age [5]; these patients are much more likely to develop severe symptoms such as those characterized by ARDS or multiple organ failure. Moreover, the significant need for early prediction of clinical progression has aroused much attention worldwide, yet it remains to be fully addressed.

Many studies highlight the potential hallmarks of COVID-19. Biochemical and radiological outcomes are the most widely recognized indexes in clinical treatment and decision making [21]; these include interleukin-6 level [22], lymphocyte count, neutrophil-to-lymphocyte ratio [23], aspartate aminotransferase level [24], and ground glass opacity on CT scan images [11,13,25]. An artificial intelligence tool focuses on early detection by screening publicly available radiological results of patients with COVID-19 with an accuracy of 86.7% [26-28]. Another recent study developed a system based on deep learning models to quantify the infectious areas in the lungs of patients with COVID-19, to predict the severity of clinical course [29]. A prognostic model based on the XGBoost algorithm with a reported accuracy greater than 90% used 3 biochemical features, including LDH, to predict the mortality rate and clinical outcomes [30], whereas another machine learning framework based on random forest, decision tree, and support vector machine used 3 different clinical features, including aminotransferase, for early prediction of clinical severity [31]. However, the accuracy of the latter model was 70%-80% when an adequate dataset was not available, as only incomplete information from 53 patients was used for the analysis. Interestingly, all published research for the prediction of clinical severity focused on either biochemical or radiological indexes only. To our knowledge, our study presents the first prognostic model using both biochemical indexes and CT scan results based on neural network and deep learning, which significantly improves the predictive capability as suggested by an AUROC score of 0.93. The limitations of this study include a limited sample size and incomplete information about the patients’ past medical history—challenges often encountered by clinicians in critical and urgent scenarios. Our future work will be focused on increasing sample size and improving data quality. Conclusions

In conclusion, the course of clinical progression might be clearer with the application of our model, and we believe our effort could provide useful opinions for early identification of severely ill patients. Thus, advanced interventions could be applied to potentially reduce mortality rates and alleviate the health care burden regarding the management of COVID-19 cases.

## Appendix

Multimedia Appendix 1 Clinical laboratory data for study patients.

Multimedia Appendix 2 Summary of all clinical laboratory features as described in Figure 4A.

## Abbreviations

ARDS: acute respiratory distress syndrome
AUROC: area under the receiver operating characteristic
CNN: convolutional neural network
CT: computerized tomography
Ct: cycle threshold
ECMO: extracorporeal membrane pulmonary oxygenation
LDH: lactate dehydrogenase
PCR: polymerase chain reaction
RT: reverse transcription
